# Medical decision making among patients with heart failure – does migration background matter?

**DOI:** 10.1186/s12875-020-01260-4

**Published:** 2020-09-13

**Authors:** Olaf von dem Knesebeck, Martin Scherer, Gabriella Marx, Sarah Koens

**Affiliations:** 1 Institute of Medical Sociology, University Medical Center Hamburg-Eppendorf, Martinistr. 52, 20146 Hamburg, Germany; 2 Department of General Practice and Primary Care, University Medical Center Hamburg-Eppendorf, Martinistr. 52, 20146 Hamburg, Germany

**Keywords:** Heart failure, Primary care, Medical decisions, Migration, Treatment, Disparities, Germany

## Abstract

**Background:**

Some studies, mainly coming from the U.S., indicate disparities in heart failure (HF) treatment according to migration/ethnicity. However, respective results are inconsistent and cannot be transferred to other health care systems. Thus, we will address the following research question: Are there differences in the diagnosis and management of HF between patients with and without a Turkish migration background in Germany?

**Methods:**

A factorial experimental design with video vignettes was applied. In the filmed simulated initial encounters, professional actors played patients, who consulted a primary care physician because of typical HF symptoms. While the dialog was identical in all videos, patients differed in terms of Turkish migration history (no/yes), sex (male/female), and age (55 years/75 years). After viewing the video, primary care physicians (*N* = 128) were asked standardized and open ended questions concerning their decisions on diagnosis and therapy.

**Results:**

Analyses revealed no statistically significant differences (*p* < 0.05), but a consistent tendency: Primary care doctors more often asked lifestyle and psychosocial questions, they more often diagnosed HF, they gave more advice to rest and how to behave in case of deterioration, they more often auscultated the lung, and more often referred to a specialist when the patient has a Turkish migration history compared to a non-migrant patient. Differences in the medical decisions between the two groups ranged between 1.6 and 15.8%. In 10 out of 12 comparisons, differences were below 10%.

**Conclusions:**

Our results indicate that are no significant inequalities in diagnosis and management of HF according to a Turkish migration background in Germany. Primary care physicians’ behaviour and medical decision making do not seem to be influenced by the migration background of the patients. Future studies are needed to verify this result and to address inequalities in HF therapy in an advanced disease stage.

## Background

There is extensive evidence of health care inequalities according to migration/ethnicity [1, 2]. Such inequalities can be defined as differences in treatment provided to members of different ethnic groups not justified by the underlying health conditions or preferences about treatment of the patient [3]. Inequalities in health care according to ethnicity/migration were found for different conditions, including heart failure (HF) [4]. It is known that health care providers can contribute to such inequalities as they hold beliefs (stereotypes) about help seekers that can influence their interpersonal behaviour as well as their decision making [1, 5].

HF is a chronic condition with a major public health relevance. Worldwide, about 26 million people are affected [6], and in Europe and the United States HF is the leading cause of hospitalizations [7]. Despite improvements in therapy and treatment of relevant risk factors, the prognosis for patients with HF is still poor, with a 5 year mortality rate of 50% [8]. In terms of HF diagnosis, primary care physicians play an important role, because they often are the first contact point for patients affected [9]. Studies analysing racial or ethnic differences in outcomes among HF patients (e.g. hospitalizations, mortality) came to inconsistent results [4, 10–13]. The same holds true for studies analysing differences in treatment. While Bagchi et al. [4] found racial and ethnic disparities in the receipt of pharmacological therapy, a study of Thomas et al. [12] showed that the provision of guideline-based care was comparable for black, Hispanic, and white patients hospitalized with HF. Most of these studies on disparities in HF care according to ethnicity/migration were conducted in the U.S.. Less is known from Germany where the migration situation [14] as well as the health care system [15, 16] is different compared to the U.S.. In Germany, about 25% of the population (about 21 million people) have a migration background, i.e. they immigrated themselves or are the offspring of immigrant parents [17]. People with a Turkish background form the largest migrant group in Germany. Generally, people with migration background are members of the German statutory health insurance system and have the same entitlements to care as non-migrants. However, they often face cultural and language barriers to health care [14].

Against this background, we will address the following research question: Are there differences in the diagnosis and management of HF between patients with and without a Turkish migration background in Germany?

## Methods

### Study design

We applied a factorial experimental design using clinically authentic video vignettes [18, 19]. The use of vignettes allow controlled manipulations while keeping other conditions like comorbidities and disease severity constant [20]. In the filmed simulated initial encounters, professional actors played patients, who consulted a primary care physician because of typical HF symptoms, including dyspnea, fatigue, peripheral edema, and cough [21]. These typical symptoms are highly suggestive for HF but not specific. Table 1 provides the information given in the vignette (for the complete script please see Additional file 1). We conducted workshops with clinical experts (cardiologists, general practitioners, and internists), HF patients, and members of the film production team, to develop the script for the videos. Reported symptoms were typical but not clear for HF, to achieve a clinically authentic scenario. The developed script was used for all vignettes, resulting in an identical dialogue in all video vignettes. In contrast, patients presented in the videos differed in terms of Turkish migration background (no/yes), sex (male/female), and age (55 years/75 years). Migration background was indicated by the name shown at the beginning of the video (Mr./Mrs. Yildiz (common Turkish name) vs. Mr./Mrs. Schmidt (common German name)), by appearance, and by Turkish accent. Since in Germany only about 30% of women with Turkish migration background wear a veil [22] we decided, to show Turkish women without a veil. Additionally, a veil would have led to a different appearance between men and women. Turkey was chosen as country of origin because people with a Turkish background are forming the largest migrant group in Germany [14]. Combination of the patient characteristics resulted in eight vignettes.

**Table 1:** Information given by the patient in the vignette

Symptom related information:	
- Fatigue	
- Dyspnea	
- Reduced exercise tolerance	
- Coughing	
- Pain in the side under exercise	
- Worsening sleeping disorder	
- Nocturia	
- Thick legs	
- Slight weight gain	
Other information:	
- Last physician consultation 5 years ago	
- Mother died a few months ago	
- No siblings	
- No family illnesses	
- Cold a few weeks ago	
- No pre-existing conditions (thyroid, blood sugar) or allergies	
- Normal to slightly increased blood pressure	

A casting was conducted to choose actors. Selected actors were instructed to portray a patient with HF symptoms in a similar way. For the purpose of an identical presentation, the first video was shown to the other actors. The part of the physician in the videos was spoken by a primary care doctor. To eliminate variation, one recording was used for all videos. A usual practice setting was chosen for the filming. The videos were shot from the perspective of the doctor who faced the patient. Thus, the patient was seen all the time. To ensure a realistic encounter, one cut videos were produced. The simulated encounter lasted about 7 min, which reflects the average duration of a consultation with a primary care doctor in Germany [23]. In addition to the name, age of the patient was shown at the beginning of the videos. Primary care physicians viewed the video vignettes on a laptop in their own consulting room during or directly after their daily consultation hours. One video was shown to each doctor. The interviewers asked the participating doctors to view the patient in the video as a patient in their own practice.

### Participants

Sample consisted of primary care doctors in Hamburg and adjacent regions of Lower Saxony and Schleswig Holstein. It was drawn from a comprehensive list of the Department of General Practice at the University Medical Center Hamburg-Eppendorf. General practitioners and internists with a maximum distance of 2 h driving time to the University Medical Center Hamburg-Eppendorf were invited to participate in a study on medical decision making in family practice per mail in a random order. To systematically investigate differences between physician attributes, the study population was stratified by a combination of physicians’ sex and length of experience as board certified general practitioner or internist (≤ 15 years or > 15 years) into four strata. To be included into the study, physicians had to work at least 20 h per week, mainly in primary care. To ascertain eligibility and willingness to participate, invited physicians were contacted by phone. Afterwards, we arranged an appointment for a 30 to 40 min individual in-person interview with the physicians in their own practice during or directly after their normal consultation hours. To acknowledge their participation and to partly offset lost revenue, participating physicians were provided a stipend.

According to a power calculation, a sample size of 128 physicians was needed to detect differences of 25% for the main effects (migration background of the patient) with a statistical power of 80% and a type-I error of 0.05. For example, if 50% of physicians would diagnose HF in case of a patient with a migration background and 75% of physicians would do so in case of a German patient, we would expect to detect a significant difference (at α = 0.05) 80% of the time. Therefore, the final sample consisted of 64 female and 64 male primary care physicians; half of them with ≤15 years of clinical experience and half of them with > 15 years of clinical experience. Each of the eight vignettes was presented four times in each stratum. Within the strata, we allocated the vignettes randomly. Among eligible doctors, the participation rate was 50.4%. According to a statement of the Ethics Committee of the Hamburg Medical Association (processing number: PV5663), no ethics approval was needed for the study. Written informed consent was obtained from each study participant.

### Measures

After viewing the video, the physicians were asked standardized and open ended questions concerning their decisions on diagnosis and therapy. To assess physicians’ information seeking behavior, the participants were asked if they would ask the person in the video any additional questions (yes/no) before deciding how to proceed and if so, what they wanted to ask (open ended). Additionally, they were asked about their tentative diagnosis (open ended) and what examinations and tests they would conduct (open ended). Moreover, we asked whether the physicians would prescribe medication (yes/no) and whether they would refer the patient to a specialist (yes/no). Finally, physicians were asked whether they would give lifestyle advice (yes/no) and if so, what they would advise (open ended).

### Analyses

The following analyses focus on differences between patients with and without a Turkish migration background. For the open ended questions, coding frames were developed. In terms of the additional questions, answers were subsumed under three thematic categories: psychosocial aspects (e.g. family or job situation), lifestyle (e.g. smoking or physical activity), and medical history (e.g. previous illnesses or medication). Number of physicians were counted who mentioned HF as a possible diagnosis and who would auscultate the lung or the heart, and who would measure blood pressure. In terms of advice giving, answers were subsumed under three thematic categories: rest, risk behaviours (e.g. smoking or malnutrition), and behaviour in case of deterioration (e.g. follow-up visit or emergency department visit).

Absolute and relative frequencies by patient migration status were calculated in contingency tables. Because of the balanced factorial design, differences due to migration are unconfounded with the other design factors (patient sex and age and physician sex and length of medical experience). To examine variations in diagnostic and therapeutic decisions by migration, Chi2-tests were used. Statistical analyses were performed using the R statistical package [24]. *P* values < 0.05 are considered statistically significant.

## Results

Table 2 shows the diagnostic and therapeutic decisions of the primary care doctors according to the migration status of the patient with HF symptoms. About half of the physicians would ask additional questions about psychosocial aspects, while about 36% would ask questions about lifestyle and 29% would address medical history. By trend, more physicians would ask questions about lifestyle and psychosocial aspects in case of a patient with a Turkish migration background, but the differences are not significant. About 81% of the physicians mention HF as possible diagnosis in case of a patient with a Turkish migration background, this rate is slightly lower in case of a German patient (not significant).

**Table 2:** General practitioner’s decisions in case of a patient with heart failure symptoms: differences according to migration background

	Total (N = 128)	Patient with migration background (*n* = 64)	Patient without migration background (n = 64)	p*
Additional questions				
Lifestyle	46 (35.9%)	26 (40.6%)	20 (31.3%)	0.27
Psychosocial aspects	63 (49.2%)	32 (50.0%)	31 (48.4%)	1.00
Medical history	37 (28.9%)	21 (32.8%)	16 (25.0%)	0.33
Diagnosis heart failure	99 (77.3%)	52 (81.3%)	47 (73.4%)	0.39
Examinations				
Auscultation of the lung	117 (91.4%)	61 (95.3%)	56 (87.5%)	0.21
Auscultation of the heart	114 (89.1%)	59 (92.2%)	55 (85.9%)	0.40
Measurement of blood pressure	105 (82.0%)	54 (84.4%)	51 (79.7%)	0.65
Prescription of drugs	4 (3.1%)	3 (4.7%)	1 (1.6%)	0.62
Advice				
Rest	28 (21.9%)	17 (26.6%)	11 (17.2%)	0.28
Risk behaviours	15 (11.7%)	7 (10.9%)	8 (12.5%)	1.00
Behaviour in case of deterioration	25 (19.5%)	16 (25.0%)	9 (14.1%)	0.18
Referral	44 (34.4%)	27 (42.4%)	17 (26.6%)	0.09

* significances (*p*-values) of Chi^2^-Test

In terms of examinations, no significant differences according to migration status of the patient occur in auscultations of the lung and the heart as well as in the measurement of blood pressure. Portion of physicians who would prescribe medication is considerably low (about 3%), with no significant differences according to migration. Slightly more physicians would give advice to rest and about behaviour in case of deterioration when the patient is a migrant from Turkey, but again differences are not significant. About one third of the physicians would refer the patient to a specialist. This rate is 15.8% higher when the patient has a migrant background (not significant).

## Discussion

In this study, we analysed differences in the diagnosis and management of HF between patients with a Turkish migrant background and non-migrant patients in Germany using a factorial experimental design with video vignettes. Results revealed no significant differences, but a consistent tendency: Primary care doctors more often asked lifestyle and psychosocial questions, they more often diagnosed HF, they gave more advice to rest and how to behave in case of deterioration, they more often auscultated the lung, and more often referred to a specialist when the patient is a migrant. Differences in the medical decisions between the two groups ranged between 1.6 and 15.8%. In 10 out of 12 comparisons, differences were below 10%. Thus, overall, there were no significant disparities in the diagnosis and management of HF between patients with and without a migration background in our study.

While this is the first study analysing disparities in HF care according to ethnicity/migration in Germany, there are some studies coming from other countries, especially from the U.S.. These studies, which are often based on retrospective analyses of medical record data, came to inconsistent results with regard to medications and guideline-based care [4, 10, 12]. In contrast, the present study surveys primary care physicians’ behaviour and medical decision making regarding various diagnostic and therapeutic aspects. This approach rests on the assumption that health care providers hold beliefs (stereotypes) about help seekers that can influence their behaviour as well as their decision making [1, 5, 25, 26]. In this regard, some studies found inequalities in doctor’s questioning and advice giving according to the ethnic background of the patient [27], while others did not find respective disparities [28, 29]. However, none of these studies addressed HF and none came from Germany. In this regard, it is important to highlight that results on treatment disparities according to migration cannot be generalized as respective studies have a specific frame of reference. They were conducted in a country with a specific health care system. There are studies indicating variations in diagnostic and therapeutic decisions between different health care systems. For example, in the U.S., more physicians would ask questions about lifestyle, order tests, and prescribe medication than in Germany when they see a patient with symptoms of a coronary heart disease [30]. Additionally, the former studies on treatment disparities according to migration focus on specific treatments, for a specific condition, and analyse specific ethnic or migrant groups. This also holds true for the findings presented here; they refer to primary care treatment decisions among HF patients with and without Turkish migrant background in Germany.

In Germany, about 25% of the population (about 21 million people) have a migration background, i.e. they immigrated themselves or are the offspring of immigrant parents [17]. This group is very heterogeneous with regard to country of origin, cultural background, and language. People with a Turkish background form the largest migrant group in Germany (about 3 million people [31]). In our study, Turkish migration background was indicated by the name shown at the beginning of the video (Mr./Mrs. Yildiz (Turkish) vs. Mr./Mrs. Schmidt (German)), by appearance, and by Turkish accent, while the dialogue was the same as in the videos with non-migrant patients. Thus, results of our study refer to those persons with a Turkish migration background who do not face language barriers. According to estimations, this applies to about two thirds of all people with a Turkish migration background in Germany [32]. Transferability of our results to other migrant groups is limited, because the level of integration might vary between migrant groups and it cannot be excluded that this may influence physicians’ medical decisions.

There are more methodological aspects that need to be considered when interpreting our findings. To meet the challenges associated with research on medical decision making, we applied a factorial design with video vignettes [18–20, 33]. The balanced factorial designs allows the estimation of unconfounded and independent differences by migration background. This is a strength of the factorial design compared to other study designs, because in other studies on medical decision making it is often difficult to control for other factors, which may also influence physicians’ decisions [33].

In terms of limitations, firstly, generalizability of our findings is limited to Hamburg and adjacent regions. Secondly, the study population is not representative for primary care physicians, as physicians were selected according to sex and length of clinical experience, to represent the stratification criteria in the factorial design. Additionally, the videos showed a first encounter between doctor and patient with HF symptoms. Therefore, it has to be considered that some physicians may have initiated some diagnostic and therapeutic steps in a subsequent consultation. A limitation of the use of filmed simulated encounters is that physicians may have answered differently in the interview compared to real patients. Nevertheless, a study on validity of clinical vignettes to examine quality of care indicates that vignettes are a valid method to assess physicians’ practice vignettes [34] and are generalizable to real patients [33]. To address the issue of external validity and to ensure the clinical authenticity of the vignettes, a number of steps were taken. Clinical experts (primary care physicians and cardiologists) as well as HF patients were involved in developing the script for the vignettes. Additionally, we employed a professional film team to produce the vignettes and chose professional actors to play the HF patients. The videos were filmed in a normal practice setting. Moreover, to foster that study participants identified themselves with the depicted situation, the encounters were filmed from the perspective of the doctor. The participants viewed the vignettes in their own consultation rooms. Furthermore, the vignettes were presented to the physicians during or directly after their practice hours, thus it is likely that they encountered real patients right before the interviews. Additionally, the physicians were asked to view the person in the video as a patient in their own practice. To prevent biased answers towards social acceptance, the participants were told that the interview was not a test but we were interested in medical decision making of primary care physicians. After they viewed the video, the physicians were asked how typical the patient in the video was for a patient in their own practice. The patient was considered very typical or rather typical by 83.6% of the respondents. Additionally they were asked how realistic the dialogue in the vignette was for an encounter in their own practice. Of the doctors, 84.4% said the dialogue was very realistic or rather realistic.

## Conclusion

Our results indicate that there are no significant inequalities in diagnosis and management of HF according to a Turkish migration background in Germany. Primary care physicians’ behaviour and medical decision making do not seem to be influenced by the migration background of the patients. Although this is a desirable result, it has to be verified in future studies. Moreover, as we focused on an initial encounter of a patient with HF symptoms, further research should address inequalities in HF therapy in an advanced disease stage.

## Supplementary information

**Additional file 1.** Complete script of the vignettes.

## Data Availability

The datasets used and/or analysed during the current study are available from the corresponding author on reasonable request.

## Abbreviations

HF: Heart Failure
US: United States
