# Supplementary material for: Medical decision making among patients with heart failure – does migration background matter?
Source: BMC Fam Pract. 2020 Sep 13;21:189. doi: 10.1186/s12875-020-01260-4 (PMC7488718; doi:10.1186/s12875-020-01260-4)
Supplement: Supplementary file 1 — Additional file 1. Complete script of the vignettes. [file 12875_2020_1260_MOESM1_ESM.pdf]

Script of the video vignettes

At the medical practice of a general practitioner. Patient (m/f) consults doctor because of unspecific complaints. Patient and doctor do not know each other. Initial contact after change of practice owner.

Doctor: Hello Mrs./Mr. Schmidt/Yildiz

Patient: Hello.

Doctor: We haven't met so far. What is the reason for your visit today?

Patient: Well, recently I have been feeling so weak all the time. Somehow tired and exhausted. Not as fit as usual.

Doctor: (affirmative) Mhm. What are your complaints? Can you specify that? Maybe you can think of examples?

Patient: I have the feeling that lately I'm out of puff very quickly. Somehow quicker than before.

Doctor: In what sort of situations does this particularly happen?

Patient: (brief pause) Well, I've never been that sporty, but I think climbing stairs has become more tiring and I need more time than I used to.

Doctor: (affirmative) Mhm… can you describe that in more detail?

Patient: Yes, well, sometimes it's even so bad that I have to take a break after a few stairs. Then it feels like after a long run. Sometimes I also have the feeling that it's really hard to breathe.

Doctor: Since when have you had these symptoms?

Patient: (thinks for a short moment) Well, a few weeks ago I had a cold. That was quite bad, I was in bed for three days. Afterwards I felt better again. (brief

pause) But I still have the feeling that it has become worse since then. Sometimes I also cough now.

Doctor: Do you have other complaints as well?

Patient: (thinks for a short moment) Hmm… I'm quite tired in the daytime and often feel weak. (brief pause) Somehow exhausted… (brief pause) Maybe it's because I don't sleep very well at night.

Doctor: (consenting) Mhm…

Patient: Yes, and sometimes there is also a sort of pressure, or stinging in the side. When I exert myself.

Doctor: Mhm, and what do you do when this pain occurs?

Patient: Then I wait a little bit, until everything calms down again, and then I can continue.

Doctor: Hmm… Exactly how long ago was that cold?

Patient: (thinks for a short moment) It was about two months ago. (brief pause) Yes, that should be it.

Doctor: And of those things you described, have you had anything like that in the past? Problems sleeping at night or problems with breathing? Or is all of this new?

Patient: Well, I'm also getting older. Once in a while, I lately did have the feeling that some things go a little slower.

Doctor: (consenting) Mhm.

Patient: Sometimes I also couldn't sleep very well, but I think it recently got worse.

Doctor: Hmm… As we meet for the first time today, I would like to get to know you a little and would ask a couple of general questions, if you don't mind.

Patient: Yes.

Doctor: How were your blood pressure values in the past?

Patient: Overall, always ok. Sometimes a little high, but always within limits I guess.

Doctor: Have you been diagnosed with any illnesses in the past?

Patient: No. As far as I know, I have nothing. But I haven't been to the doctor for quite a while.

Doctor: When was the last time?

Patient: (thinking) About five years ago. I am quite healthy in general.

Doctor: Have there ever been abnormalities with blood sugar?

Patient: (thinks) Well, I cannot say for sure, but I don't think I ever had problems with blood sugar.

Doctor: And what about the thyroid gland? Has there been anything there?

Patient: (thinks briefly) No, I don't think so.

Doctor: Do you regularly take any medication at the moment?

Patient: No.

Doctor: Is there medication you cannot tolerate? Do you have any allergies?

Patient: Not that I know of.

Doctor: Have you travelled recently?

Patient: No, not recently. The last time about a year ago.

Doctor: When was your last check-up? I mean blood test, urine and a general physical examination?

Patient: Hmm, I cannot say for sure. Definitely not since my last visit to the doctor.

Doctor: What about known illnesses in your family? With your parents and siblings?

Patient: My parents were always in good health, as far as I now. (brief pause) My father had a road accident and died many years ago. My mother died a few months ago. That was very difficult for me. (lowers his/her gaze, seems sad). She was very old but actually healthy.

Doctor: I'm very sorry. (brief pause) Do you have any brothers or sisters?

Patient: No.

Doctor: Let's get back to your current complaints. You said that recently, you do not sleep very well. How does this express itself? Do you have trouble falling asleep? Or do you rather wake up in the night?

Patient: Falling asleep is not a problem. Anyway, I'm always quite tired. I lie down and after 10 minutes I fall asleep. But most of the time I wake up in the night.

Doctor: Why do you wake up?

Patient: Hmm… I cannot tell for sure. Sometimes I have a sort of a restlessness. Sometimes I also suddenly woke up with a start. Then I had to take a deep breath and then it was ok again.

Doctor: Do you recently wake up more often than in the past?

Patient: Yes, much more often.

Doctor: Do you then have to go to the toilet?

Patient: Yes, in most cases I then have to go to the toilet. Sometimes really urgent.

Doctor: Lately, do you drink more than in the past? For example before going to bed?

Patient: No, not really.

Doctor. And what about in the daytime? You said you often feel tired and exhausted. Do you have any other complaints?

Patient: (thinks) Well, when I'm out and about for a longer period of time, my legs hurt. Then I have the feeling they are thicker than usual.

Doctor: Have you recently lost weight?

Patient: No. I rather think I might have gained a little weight. (brief pause) It's funny, because I actually have less appetite and I also eat less than I used to. Nevertheless and funnily enough, I often feel so full.

Doctor: Is there something currently on your mind that stresses you? Are you worried about something?

Patient: (thinks for a short time) No, (brief pause), only what I just mentioned. Other than that actually nothing (a little thoughtful).

Fade out.
